# Genome reduction in *Paenibacillus polymyxa* DSM 365 for chassis development

**DOI:** 10.3389/fbioe.2024.1378873

**Published:** 2024-03-28

**Authors:** Giulia Ravagnan, Janne Lesemann, Moritz-Fabian Müller, Anja Poehlein, Rolf Daniel, Stephan Noack, Johannes Kabisch, Jochen Schmid

**Affiliations:** ^1^ Institute of Molecular Microbiology and Biotechnology, University of Münster, Münster, Germany; ^2^ Institute of Bio-and Geosciences, IBG-1: Biotechnology, Forschungszentrum Jülich GmbH, Jülich, Germany; ^3^ Department of Genomic and Applied Microbiology and Göttingen Genomics Laboratory, Institute of Microbiology and Genetics, Georg-August-University Göttingen, Göttingen, Germany; ^4^ Department of Biotechnology and Food Science, Norwegian University of Science and Technology, Trondheim, Norway

**Keywords:** *Paenibacillus polymyxa*, genome reduction, chassis, BGCS, 2,3-butanediol

## Abstract

The demand for highly robust and metabolically versatile microbes is of utmost importance for replacing fossil-based processes with biotechnological ones. Such an example is the implementation of *Paenibacillus polymyxa* DSM 365 as a novel platform organism for the production of value-added products such as 2,3-butanediol or exopolysaccharides. For this, a complete genome sequence is the first requirement towards further developing this host towards a microbial chassis. A genome sequencing project has just been reported for *P. polymyxa* DSM 365 showing a size of 5,788,318 bp with a total of 47 contigs. Herein, we report the first complete genome sequence of *P. polymyxa* DSM 365, which consists of 5,889,536 bp with 45 RNAs, 106 tRNAs, 5,370 coding sequences and an average GC content of 45.6%, resulting in a closed genome of *P. polymyxa* 365. The additional nucleotide data revealed a novel NRPS synthetase that may contribute to the production of tridecaptin. Building on these findings, we initiated the top-down construction of a chassis variant of *P. polymyxa*. In the first stage, single knock-out mutants of non-essential genomic regions were created and evaluated for their biological fitness. As a result, two out of 18 variants showed impaired growth. The remaining deletion mutants were combined in two genome-reduced *P. polymyxa* variants which either lack the production of endogenous biosynthetic gene clusters (GR1) or non-essential genomic regions including the insertion sequence IS*Pap1* (GR2), with a decrease of the native genome of 3.0% and 0.6%, respectively. Both variants, GR1 and GR2, showed identical growth characteristics to the wild-type. Endpoint titers of 2,3-butanediol and EPS production were also unaffected, validating these genome-reduced strains as suitable for further genetic engineering.

## Introduction

In the rapidly evolving landscape of biotechnology, the demand for new and unconventional microbial systems with sophisticated properties compared to established model organisms greatly increased (Blombach et al., 2021). Optimisations, to reach industrially relevant product titers and efficiencies, are of the highest interest. Genome reduction, using top-down strategies, is regarded as a valuable tool for the construction of future chassis for bio-based productions (LeBlanc and Charles, 2022). Many studies have shown several advantages such as improved product formation, minimised wasted energy, reduced complexity and simplified metabolic background, facilitating product identification or purification. The deletion of 15 native antibiotic gene clusters in *Streptomyces albus* improved the production of five heterologous expressed biosynthetic gene clusters of around 2-fold compared to the parent strain (Myronovskyi et al., 2018). In *Pseudomonas chlororaphis* the deletion of five antibiotic gene clusters and other 17 non-essential regions resulted in the increased production of the native phenazines, such as 4.4-fold higher 2-hydroxyphenazine (Shen et al., 2017). A genome-reduced *Bacillus subtilis* was engineered for the production of guanosine and thymidine showing 4.4-fold and 5.2-fold increases, respectively (Li et al., 2016). The latter study demonstrates how genome-reduced strains can be further genetically engineered for a specific product showing better traits than the parental strain. Unthan et al. also initially constructed several *Corynebacterium glutamicum* streamlined chassis which displayed a normal growth behaviour (Unthan et al., 2015). Further modifications resulted in C1* chassis that was then successfully engineered for the production of indole due to its higher tolerance towards this product compared to the WT (Baumgart et al., 2018; Mindt et al., 2022; Mindt et al., 2023).

Another advantage of genome reduction is the enhancement of genetic stability through the deletion of insertion sequences (IS) and prophages (Baumgart et al., 2013; Choi et al., 2015; Umenhoffer et al., 2017). Insertion sequences are well-known mobile elements which encode for transposase genes, capable of spreading to high relevance within a genome, strongly impacting genomic structure and function (Siguier et al., 2015). Choi et al. improved a *C. glutamicum* production strain by deleting two active IS elements, increasing the productivity and yield of poly (3-hydroxybutyrate) and γ-aminobutyrate (Choi et al., 2015). The deletion of six copies of IS1236 in *Acinetobacter baylyi* enabled a 21-fold reduction of mutation rates and increased transformability (Suárez et al., 2017). Similarly, *Magnetospirillum gryphiswaldense* deprived of IS elements displayed increased genetic stability and resilience, while maintaining normal growth behaviour and magnetosome production (Zwiener et al., 2021).

*Paenibacillus polymyxa* is a Gram-positive, facultative anaerobic, non-pathogenic soil bacterium, mostly known for its use as a biocontrol agent and biofertilizer (Jeong et al., 2019). In particular, *P. polymyxa* DSM 365 has great potential as a new unconventional cell factory since various efficient tools for genetic and genomic engineering are established and it offers a broad native metabolic capability (production of antimicrobials and commercially relevant compounds, *e.g., R,R-*2,3-butanediol and exopolysaccharides) (Rütering et al., 2016, 2017; Schilling et al., 2020; Schilling et al.2021; Schilling et al., 2022; Schilling et al., 2023; Blombach et al., 2021; Meliawati et al., 2022b; Meliawati et al., 2022a). To significantly advance research towards a future chassis organism the completion of the published draft genome sequence was essential. Especially, for the targeted design of a streamlined genome by the use of the CRISPR-Cas technology.

In this study, we completed the genome sequence of *P. polymyxa* DSM 365 which enabled the identification of an unknown Non-Ribosomal Peptide Synthase (NRPS) and helped define deletion targets for the final construction of two genome-reduced strains. This research aimed to construct two platform strains for biotechnological application with reduced metabolic complexity and genetic instability while maintaining the physiological behaviour of the wild-type (WT). We first investigate the physiological effect of deleting single targets, through the assessment of the growth rate, an easily measurable parameter directly indicating unchanged or altered biological fitness. Finally, we combined the best-performing mutants in two genome-reduced strains, GR1 and GR2. In the controlled environment of a bioreactor, both strains showed a growth and metabolic behaviour comparable to the WT, validating them as future platform strains for industrial bioprocess development.

## Materials and methods

### Genome sequencing

*P. polymyxa* DSM 365 was obtained from the German Collection of Microorganisms and Cell Culture (DSMZ), Germany. Genomic DNA was extracted from the strain cultivated at 30°C 250 rpm in LB medium (10 g L^-1^ peptone, 5 g L^-1^ yeast extract, and 5 g L^-1^ NaCl) using the DNeasy Blood and Tissue Kit (Qiagen). Nanopore sequencing was performed on the MinION with the ligation sequencing kit SQK-LSK109 and EXP-NBD104 without pore selection (Oxford Nanopore Technologies, Oxford, UK) and Illumina sequencing using the Illumina MiSeq kit v3 (Illumina, San Diego, USA). The sequencing data was assembled using Unicycler v0.4.9 (Wick et al., 2017). The genome was then annotated by RAST server online (Aziz et al., 2008) and further analysed using the computational tools: RNAmmer and tRNAscan-SE to identify coding RNAs, BLAST with Rfam database for non-coding RNAs, antiSMASH to determine biosynthetic gene clusters (BGCs), PHASTER to annotate prophage sequences, ISFinder to annotate insertion sequences and finally Islandviewer to annotate genomic islands (GIs) (Lowe and Eddy, 1997; Siguier et al., 2006; Lagesen et al., 2007; Gardner et al., 2009; Arndt et al., 2016; Blin et al., 2021). Manual curation and homology searching with other *Paenibacillus* species helped to define specific direct repeats (DRs) as well as inverted repeats (IRs) of each IS element.

### Strains

Plasmid cloning and multiplication were performed in *Escherichia coli* turbo (New England Biolabs, USA) or TOP10 (Invitrogen). *E. coli* S17-1 (ATCC 47055) was used as a conjugative donor strain to mediate the transfer of plasmid DNA to *P. polymyxa*. The strains were cultivated in LB medium. For plate media, an additional 1.5% of agar were used. Whenever necessary, the media were supplemented with 50 μg mL^-1^ neomycin and 40 μg mL^-1^ polymyxin. The strains were stored as cryo-cultures in 24% glycerol and kept at −80 °C for longer storage.

### Plasmid construction

All plasmids generated in this study were constructed by the isothermal assembly and transferred to *P. polymyxa* via conjugation, as previously described (Meliawati et al., 2022b). The genome modifications were facilitated by the CRISPR-Cas9-based system developed for *P. polymyxa* (Rütering et al., 2017). In brief, the guide RNA (gRNA) for targeted genome editing was designed by using the Benchling CRISPR design tool. Approximately 0.4–1 kb homologous regions upstream and downstream of the targeted sites were amplified from genomic DNA (gDNA) of *P. polymyxa* and provided as a repair template. After transformation, screening of the colonies was performed by colony PCR using GoTaq Polymerase (Promega). Subsequently, the plasmids were isolated and sent for sequencing to confirm the correct assembly. Next, chemically competent *E. coli* S17-1 was transformed with the correctly assembled plasmid for the following conjugational transfer to *P. polymyxa*. Synthesis of oligonucleotides and sequencing analysis were performed by Microsynth (Germany). In silico plasmid cloning was planned by the use of Geneious prime version 2020.2.5. The list of oligonucleotides and plasmids used in this study is provided in Supplementary Table S1, S2, respectively.

### Conjugation

Conjugation was performed between *P. polymyxa* (recipient strain) and *E. coli S17-1* harboring the plasmid of interest (donor strain). The cryo-cultures of both strains were streaked on LB plates containing suitable antibiotics, and if necessary, following overnight liquid cultures were prepared from the colonies obtained on the plates. The overnight cultures were diluted 1:100 in 3 mL LB media, with or without antibiotics, in 13 mL plastic culture tubes. Incubation was performed at 37°C, 250 rpm, for 4 h. Subsequently, 900 µL of the recipient strain was heat shocked at 42°C for 15 min and mixed with 300 µL of the donor culture. The mixture was centrifuged at 8,000 g for 3 min and the supernatant was discarded. The cell pellet was resuspended in 100 µL of LB media and the resuspension was dropped onto an LB agar plate. After overnight incubation at 30°C, the cells were scraped off the plate and resuspended in 100 µL of LB media. Afterwards, the resuspension was plated on an LB agar plate containing 50 μg mL^-1^ neomycin and 40 μg mL^-1^ polymyxin. If necessary, the resuspension was diluted with appropriate dilution to obtain countable colonies on the plates. The plate was incubated at 30°C for 48 h to obtain *P. polymyxa* exconjugants. Screening of the exconjugants was performed by colony PCR and sequencing of the resulting DNA fragments. Plasmid curing was performed by 1:100 diluted subcultivations every 24 h at 37°C.

### Quantitative RT-PCR

RNA extraction of positive samples of the GFP fluorescence assay as well as the butanediol fermentation processes was performed using the Aurum Total RNA Mini Kit (BioRad) according to the manufacturer’s instructions. The synthesis of cDNA was conducted using iScript reverse transcriptase (BioRad) by use of 1 μg of total RNA as a template. The qPCR reactions were performed in triplicate in a CFX-96 thermocycler using SsoAdvanced universal SYBR Green Supermix (BioRad) using 5 ng of cDNA as a template in 10 μL reaction volume. Negative controls without reverse transcriptase during cDNA synthesis were used to evaluate the absence of gDNA contaminations. The relative gene expression levels were calculated based on the ΔΔCq method and *gyrA* was used as a reference gene (Livak and Schmittgen, 2001). After qPCR, a melting curve analysis was performed to confirm the presence of a single PCR product for each target. The designed primers were analyzed by the OligoAnalyzer Tool (IDT) to avoid hairpin formation and self- and heterodimer formation with free energy values of more than 10 kcal mol^−1^. The oligonucleotides used for qPCR experiments are listed in Supplementary Table S1.

### Cultivation in microtiterplates

Growth analysis for evaluation of single knock-out mutants was performed in FlowerPlates in a BioLector (m2p-labs, Germany) with a total volume of 1 mL. A single colony was used for the inoculation of 4 mL preculture in 24 deep well plates and grown overnight at 1,000 rpm and 30°C in CASO Broth medium (17 g L^-1^ casein peptone, 3 g L^-1^ soy peptone, 5 g L^-1^ NaCl, 2.5 g L^-1^ K_2_HPO_4_, 2.5 g L^-1^ glucose). The next day a 1:5 dilution was performed, with a following 2 h incubation at the same conditions, OD_600_ measurements were then performed to start growth experiments in FlowerPlates at 1,000 rpm, 95% humidity, 30°C, and backscatter (BS) gain 20 with a starting OD_600_ of 0.1. For subsequent data analysis, each BS curve was first blanked by its initial value.

### Batch fermentation

Batch fermentations were conducted in 1 L parallel DASGIP bioreactors (Eppendorf, Germany) with an initial volume of 550 mL fermentation medium from Schilling et al. (Schilling et al., 2020). A single colony from a freshly streaked plate was used to inoculate 100 mL in the pre-culture fermentation medium grown at 30^°^C with 160 rpm. After overnight incubation, the bioreactors were inoculated from these precultures growing exponentially to an initial OD_600_ of 0.1. Samples were taken regularly over the course of 48 h for OD_600_, CDW and metabolite analysis. Fermentation was performed at 35 °C and constant aeration of 0.5 vvm with constant stirring at 300 rpm. The pH value was maintained at 6.0 and automatically adjusted with 2 M NaOH or 1.35 M H_3_PO_4_. One % antifoam B (Sigma-Aldrich, Germany) was used for foam control.

### Calculation of specific growth rate

Maximum specific growth rates during exponential growth were calculated from the slope of the semi-logarithmic plot of BS or OD_600_ against time. The obtained growth rates for all strains were analysed for significant changes compared to reference organisms (WT) with the two-sample t-test (*p < 0.05, ***p < 0.001).

### Analytical methods

Cell growth was determined by measuring optical density at 600 nm (OD_600_). For cell dry weight (CDW) measurements, 10 mL of fermentation broth was centrifuged at 5,000 g for 20 min in already dried and pre-weight tubes. Cell pellets were washed twice with 0.9% NaCl and dried overnight at 100°C. Culture supernatants were obtained by centrifugation of 1 mL culture sample at 14,000 g for 5 min. After centrifugation, the resulting supernatant was filtered through 0.2 μm PTFE filters and utilized for HPLC-UV-RID analysis. Glucose and product concentrations were determined via a HPLC-UV-RID system (Dionex, USA) equipped with Rezex ROA-H^+^ organic acid column (300 mm × 7.8 mm, Phenomenex, USA). Column temperature was set to 70°C and 2.5 mM H_2_SO_4_ was used as the mobile phase with a flow rate of 0.5 mL min^-1^ and 40 min run time. EPS concentrations were determined by centrifugation (8.000 g, 15 min) of 100 mL aliquots of fermentation broth at the end of the cultivation process. The supernatant was slowly poured into 200 mL of isopropanol while stirring. Precipitated EPS was collected, and dried over-night in a vacuum oven (45°C and 40 mbar) (Heraeus, Germany) and following gravimetric weight determination.

## Results and discussion

### Genome sequencing of *P. polymyxa* DSM 365

The genome consists of a 5,889,536 bp chromosome, with a GC content of 45.6%. The assembled data showed an average coverage of 210.5-fold. Annotation of the genome revealed a total of 5,370 genes. Previously, the genome of *P. polymyxa* DSM 365 was also sequenced by Neng-Zhong Xie et al. and Kumar et al. Both sequences are presented as 84 and 47 contigs, respectively (Xie et al., 2015; Kumar and Ujor, 2022). The most recently published genome, consisting of 47 contigs, was compared to our sequenced genome. This comparison showed that our latest genome sequence has a higher number of base pairs which is 5,889,536 bp, compared to 5,788,318 bp (Table 1). Many of the contigs of the genome published by Kumar et al. are interrupted by repetitive sequences and a total of 101,218 bp is missing. These additional sequences include the repetitive sequences of rRNAs, tRNAs, NRPSs and IS. This allows the number of the rRNAs to be corrected to 42, compared to the 49 sequences reported previously. Such an overestimation is linked to poorly assembled gene sequences which are spread on different contigs, thus artificially increasing the number of gene copies. The complete genome contains 14 23S, 14 16S and 14 5S rRNA sequences, precisely 42 in total. The number and diversity of tRNAs was also underestimated: a total of 106 tRNAs were found, compared to 104 in the genome published by Kumar et al. In addition, about 23,300 bp of one of the NRPS of the tridecaptin cluster (*triD*) is missing, together with the sequence of another unknown NRPS of 42,306 bp (Lohans et al., 2014). This unknown NRPS sequence (*unrps*) piqued our curiosity and we analysed it further.

**TABLE 1:** Comparison of genomic features of *P. polymyxa* DSM 365 genome of this study vs. *P. polymyxa* DSM 365 genome published by Kumar et al. (Kumar and Ujor, 2022).

Features	Chromosome	
	*P. polymyxa* DSM 365 Closed genome this study	*P. polymyxa* DSM 365 Genome published by Kumar et al
Genome size (bp)	5,889,536	5,788,318
Number of contigs	1	47
G + C content (%)	45.6	45.5
tRNAs	106	104
rRNAs	42	49
ncRNA	3	3
Accession number	CP141264	JAKVDC010000000

### Genome mining studies, unravelling a potential role of the unknown NRPS

**FIGURE 1:**
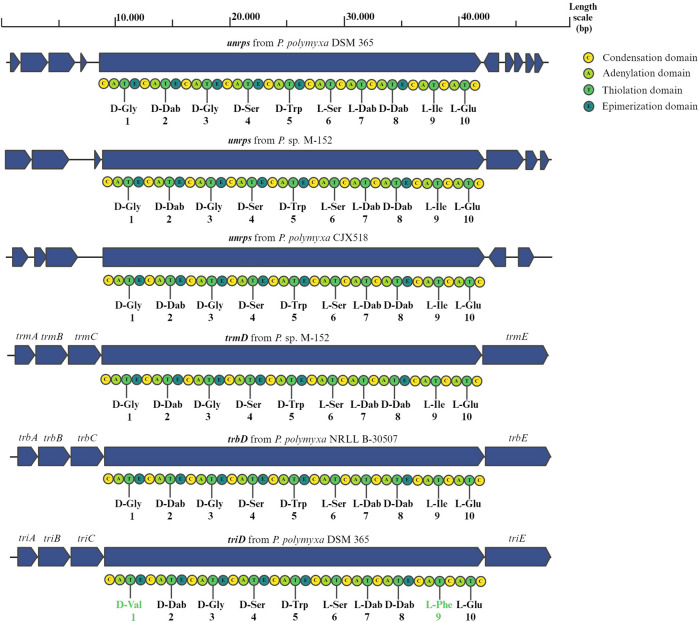
Organization of the nearby genes of the new NRPSs from the genomes of *P. polymyxa* DSM 365, *P.* sp. M-152, *P. polymyxa* CJX518 and tridecaptin cluster from *P.* sp. M-152, *P. polymyxa* NRLL B-30507, and *P. polymyxa* DSM 365. The sequences of their peptide moieties were predicted by bioinformatic evaluation of the A-domains of their amino acid specific modules.

The gene for the putative NRPS shows protein identities of 90.2% and 100% coverage with TriD, the main NRPS of the tridecaptin cluster. The sequence prediction from the adenylation domains of the unknown NRPS suggests the decapeptide D-Gly-D-Dab-D-Gly-D-Ser-D-Trp-L-Ser-L-Dab-D-Dab-L-Ile-L-Glu, which is the same expected decapeptide of the NRPS of tridecaptin M (TrmD) from *Paenibacillus* sp. M-152 and of the NRPS of the tridecaptin B (TrbD) from *Paenibacillus polymyxa* NRRL B-30507 (Cochrane et al., 2015; Jangra et al., 2019). The tridecaptin clusters (A, B and M) are known to present five genes encoding for the synthesis and secretion of the active peptide: *triA* (a putative thioesterase), *triB* and *triC* (ABC transporters), and *triD* and *triE* (NRPSs) (Lohans et al., 2014; Cochrane et al., 2015; Jangra et al., 2019). The *triD* gene encodes for a protein that assembles the first 10 amino acids of tridecaptin. The biosynthesis is then completed with the addition of the last three amino acids by TriE due to the presence of a thioesterase domain (TE). However, the NRPS we identified does not have this cluster of genes nearby. We therefore compared the genome with the genomes of other *Paenibacilli*, which also have the tridecaptin cluster to analyse if they also have a similar NRPS. To do this, we blasted our NRPS protein against the genomes of *P.* sp. M-152, *Paenibacillus polymyxa* CJX518, *Paenibacillus polymyxa* HY96-2 and *Paenibacillus polymyxa* E681 (Camacho et al., 2009). In the first two strains, but not in *P. polymyxa* HY96-2 and *P. polymyxa* E681, we identified an NRPS with 92.9% protein identities with 100% coverage, an even higher percentage compared to TriD. However, we did not find any conserved genes in the vicinity of the different synthetases, suggesting that there is no nearby gene involved in the synthesis or excretion of the molecules. Furthermore, these NRPSs do not show a thioesterase domain at their 3′-end (Figure 1). Consequently, the termination of the biosynthesis of the peptide is accomplished by either an external thioesterase free-enzyme or by TriE. In the latter case, these NRPS sequences, which present a high similarity with both TriD and TrmD and the synthesized decapeptide, might represent an additional copy required for the synthesis of tridecaptins contributing to tridecaptin´s diversity. To strengthen this hypothesis, we have compared the module 10 of the unknown NRPS with the one of TriD of *P. polymyxa* DSM 365 and we have found 93.1% protein identities with 100% coverage. Based on that, we consider the modules to be highly similar. To verify the theory of the unknown NRPS playing a role in tridecaptin synthesis, further studies should elucidate the effect of the deletion of the unknown NRPS more in general and on tridecaptin production.

### Determination of deletion targets and genome reduction strategies

**FIGURE 2:**
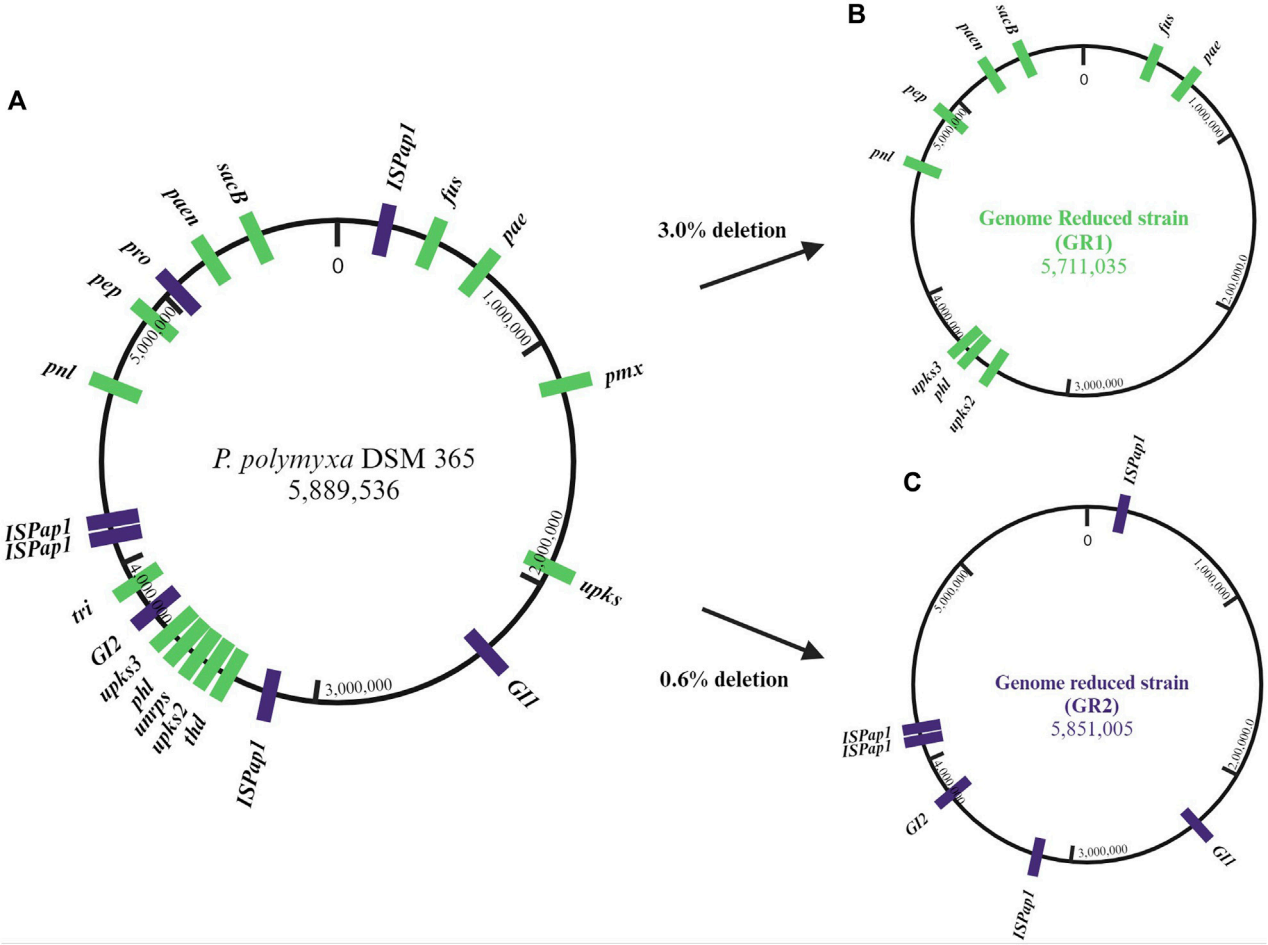
**(A)** Circular genome map of *P. polymyxa* DSM 365, targeted deletion regions of secondary metabolites were marked in green and targeted deletion of genomic islands (GI), prophage and insertion sequences (IS) were marked in violet on the physical localizations of the chromosome. **(B)** The genome-reduced strain GR1 shows the sequential deletion of several biosynthetic gene clusters (BGC). **(C)** The genome-reduced strain GR2 shows the sequential target deletions of two GIs and four copies of *ISPap1*.

The closed genome sequence was analysed by the use of different bioinformatic tools (antiSMASH, PHASTER, IslandViewer and ISfinder) (Siguier et al., 2006; Arndt et al., 2016; Bertelli et al., 2017; Blin et al., 2021). The predictions indicate the presence of 13 antibiotic biosynthetic gene clusters (BGCs) of which 4 are unknown, one incomplete prophage region, 16 genomic islands (GIs) and six different insertion sequences (IS) of which IS*Pap1* has six copies around the genome. This corrects a previous pangenome analysis in which *P. polymyxa* DSM 365 was reported to not include the paenilipoheptin and paenilan clusters (Wang et al., 2020). All antibiotic BGCs were selected as possible targets, aside bacillibactin due to its known role in iron uptake (May et al., 2001; Barona-Gómez et al., 2006). Additionally, the two known genomic regions encoding for the exopolisaccharides (EPS), paenan and levan, were also selected (Rütering et al., 2016, 2017; Schilling et al., 2022; 2023). The selected secondary metabolite encoding cluster, the incomplete prophage region, the EPS encoding sequences and the genomic islands were deleted individually and the resulting strain variants were characterised concerning fitness (Table 2). Following this, the first strategy for a genome-reduced strain variant was followed by consecutively deleting the EPSs and antibiotic targets to diminish the native metabolite background which competes for precursors and energy, leading to the designated GR1 variant (Figure 2). Precisely, paenan (32.8 kb), levan (1.60 kb, *sacB* gene), paenibacillin (11.9 kb), paenilan (12.5 kb), unknown pks 2 (40.2 kb), unknown pks 3 (24.5 kb), fusaricidin (31.0 kb), paenilipoheptin (17.4 kb, a part of this BGC) and paenicidin (6.46 kb, a part of this BGC) clusters were deleted. The second strategy aimed at generating an ISPap1-free mutant to enhance its genomic stability, which led to the designated GR2 variant (Figure 2). One copy of IS*Pap1* and of another IS, located in the same GI, were deleted as a single deletion (GI1). Another copy of the insertion sequence IS*Pap1* was identified in another genomic island. For this reason, this complete sequence was also deleted at once (GI2). Before deletion, both genomic islands were evaluated to not contain putative essential genes by analysing the whole-genome sequence by the use of DEG10 (Database of Essential Genes) (Luo et al., 2014). The open reading frames which were predicted in the GIs include mainly hypothetical proteins, possibly transcriptional regulators, and hydrolases. Based on DEG10 results, BLAST, and PFAM analysis, we hypothesized that the selected regions will not affect the fitness of our strain. The remaining four copies of IS*Pap1* were then stepwise deleted by use of the CRISPR-Cas9 approach (Table 3). Detailed information such as the coordinates of each of the deletions are given in Supplementary Table S3.

**TABLE 2:** List of the most important bacterial strains and strain variants used in this study.

Strains	Genotype and description	Source or reference
*E. coli* TOP10	F-mcrAΔ(*mrr-hsdRMS-mcrBC*) Φ80*LacZ*ΔM15 Δ*Lac*X74 *rec*A1 *ara*D139 Δ(*araleu*) 7697*gal*U *gal*K *rps*L (StrR) *end*A1 *nup*G	Invitrogen
*E. coli* Turbo	F' *proA* ^*+*^ *B* ^*+*^ *lacI* ^*q*^ ∆*lacZM15/fhuA2* ∆(*lac-proAB) glnV galK16 galE15* * * *R* (*zgb-210::Tn10*)TetS *end*A1 *thi*-1 ∆(*hsdS-mcrB*)5	NEB
*E. coli* S17-1	Conjugation strain; recA pro hsdR RP42Tc:Mu-Km:Tn7 integrated into the chromosome	ATCC 47055
*P. polymyxa* DSM 365	Wild-type	DSMZ
Single deletion strains		
S45115	Δ*pmx,* polymyxin	Meliawati et al. (2022b)
S45126	Δ*fus,* fusaricidin	Meliawati et al. (2022b)
S45143	Δ*paen,* paenibacillin	This study
S45144	Δ*pnl,* paenilan	This study
S45145	Δ*pep*, paenan	Meliawati et al. (2022b)
S45146	Δ*sacB*, levan	Schilling et al. (2020)
S45150	Δ*pro*, prophage	This study
S45151	Δ*unrps*, unknown NRPS	This study
S45166	Δ*GI1*, genomic island 1	This study
S45167	Δ*GI2,* genomic island 2	This study
S45175	Δ*upks2*, unknown pks 2	This study
S45195	Δ*tri*, tridecaptin	This study
S45196	Δ*upks3,* unknown pks 3	This study
S45209	Δ*upks*, unknown pks	This study
S45244	Δ*phl*, a part of the cluster paenilipoheptin	This study
S45245	*Δthd*, thermoactinoamide	This study
S45246	Δ*GI1*Δ*GI2*Δ*ISPap1-3*Δ*ISPap1-1* Δ*ISPap1-5*	This study
S45248	*Δpae,* a part of the cluster of paenicidin	This study
Genome-reduced strain 1		
GR1	Δ*pep*Δ*sacB*Δ*paen*Δ*pnl*Δ*upks2*Δ*upks3* Δ*fus*Δ*phl*Δ*pae*	This study
Genome-reduced strain 2		
GR2	Δ*GI1*Δ*GI2*Δ*ISPap1-3*Δ*ISPap1-1* Δ*ISPap1-5*Δ*ISPap1-6*	This study

**TABLE 3:** Detailed information of deleted genomic regions of the constructed genome-reduced mutant strains of *P. polymyxa* via the two parallel strategies.

Mutants	Deletion region	Genome size (bp)	Cumulative deletion size (bp)
*P. polymyxa* wt	-	5,889,536	-
Genome-reduced strain variant 1 (**GR1**)	Sequential deletions of BGCs		
	Δ*pep*	5,856,690 (99.44%)	32,846
	Δ*sacB*	5,855,095 (99.42%)	34,441
	Δ*paen*	5,843,195 (99.22%)	46,341
	Δ*pnl*	5,830,722 (99.01%)	58,814
	Δ*upks2*	5,790,497 (98.32%)	99,039
	Δ*upks3*	5,765,952 (97.90%)	123,584
	Δ*fus*	5,734,877 (97.37%)	154,619
	Δ*phl*	5,717,489 (97.08%)	172,007
	Δ*pae*	5,711,035 (96.99%)	178,461
Genome-reduced strain variant 2 (**GR2**)	Sequential deletions of GIs and IS		
	Δ*GI1*	5,870,461 (99.67%)	19,075
	Δ*GI2*	5,857,445 (99.46%)	32,091
	Δ*ISPap1-3*	5,855,857 (99.42%)	33,679
	Δ*ISPap1-1*	5,854,279 (99.40%)	35,257
	Δ*ISPap1-5*	5,852,682 (99.37%)	36,854
	Δ*ISPap1-6*	5,851,005 (99.36%)	38,531

### Growth characterization of single deletion variants

**FIGURE 3:**
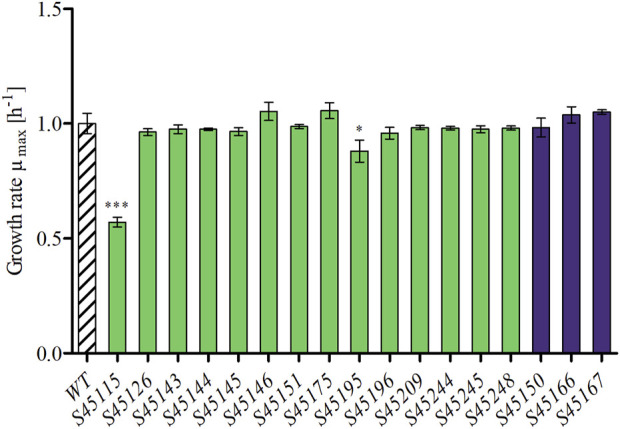
Maximum growth rates (μ_max_) of single mutants of *P. polymyxa* in CASO broth medium (n ≥ 4). Strains with significant changes in maximum growth rate compared to the reference (WT) were determined via two sample t-test (*p < 0.05, ***p < 0.001) and are marked by asterisks.

Each single mutant was first cultivated in CASO broth and characterised for putative effects on biological fitness via assessment of the maximum specific growth rate (μ_max_), which was determined from multiple replicates (n ≥ 4). Significantly altered μ_max_ values were evaluated via the two-sided t-tests (*p* < 0.01) by comparing each mutant with the *P. polymyxa* WT (Figure 3). For the WT, a μ_max_ of 1.00 ± 0.04 h^–1^ was determined, and the values for the single knockouts ranged between 0.53 ± 0.02 h^–1^ (S45115) and 1.04 ± 0.04 h^–1^ (S45167) (Figure 3). Strains S45115 (0.53 ± 0.02 h^–1^) and S45195 (0.88 ± 0.04 h^–1^), with deletions in the polymyxin and tridecaptin cluster, respectively, grew significantly slower than the WT and therefore were not considered further as deletion targets. Instead, all other mutants maintained the growth behaviour at the wild-type level. All growth curves of both the WT and the mutants are presented in Supplementary Figure S1.

Many genome reduction strategies have targeted the deletion of various antibiotics encoding BGCs, resulting in unaffected growth behaviour of these deletions (Gomez-Escribano and Bibb, 2011; Shen et al., 2017; Myronovskyi et al., 2018; Ahmed et al., 2020). Exceptions are reported for the deletion of siderophores and a *Schlegelella brevitalea* genome-reduced strain variants devoid of five–7 BGCs (DC5-DC7) (Barona-Gómez et al., 2006; Liu et al., 2021). In this study, strains S45115 and S45195, with the deletions of polymyxin and tridecaptin encoding clusters respectively, also represent one of these exceptions. Both clusters include genes which encode for NRPS and transporters for excretion of these specific products (Lohans et al., 2014; Tambadou et al., 2015). Shaheen et al. deleted the two genes *pmxC* and *pmxD*, each encoding for a transporter in the polymyxin cluster and showed a decreased production of both polymyxin and fusaricidin, demonstrating that both antibiotics are still exported by other transporters. Yet, no additional information on growth behaviour and strain fitness was presented by the authors (Shaheen et al., 2011). The deletion of the single NRPS of the two clusters (*pmxE* and *triD*) did not result in significant growth defects for these variants (Kim et al., 2014). This suggests that both polymyxin and tridecaptin play an essential role for growth under the applied conditions or that the deletion might have indirectly caused an impairment. Further experiments should elucidate these possibilities.

### qPCR to check the transposase of IS*Pap1* transcriptional expression

**FIGURE 4:**
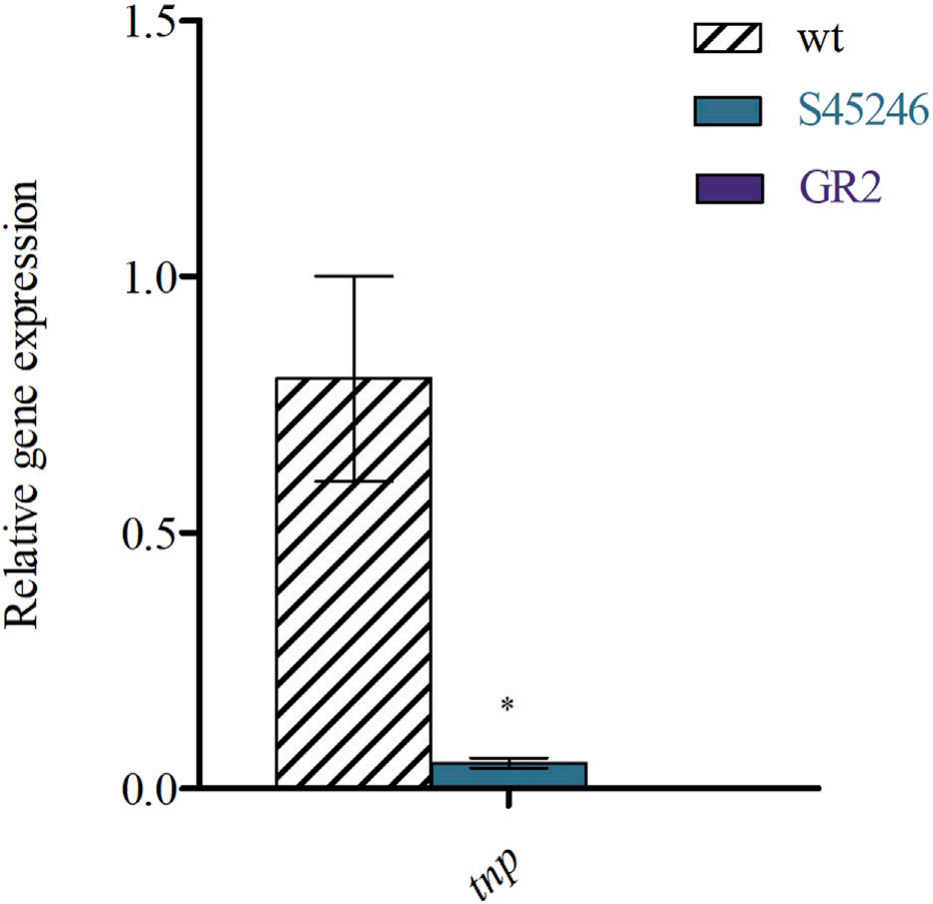
RT-qPCR of IS*Pap1* transposase (*tnp*) gene levels relative to the housekeeping gene *gyrA* in the WT, S45246 and GR2. *p < 0.05 was determined via two sample t-test of three biological replicates grown in CASO broth.

As previously mentioned, genome sequencing revealed the presence of six copies of the insertion sequence IS*Pap1*. Before deleting all these copies, which results in the strategy for GR2, we analysed if the transposase (*tnp*) is actually transcriptionally expressed. For this, we performed qPCR by targeting the first part of the transposase. As shown in Figure 4, we were able to demonstrate its expression and consequently assume a possible activity, which led us to proceed with the deletions. Studies of other transposases also show transcriptional expression and a related transposition activity at different conditions (Lee et al., 2019; Kirsch et al., 2023). Yet, further studies should elucidate if IS*Pap1* is truly active (Tavakoli and Derbyshire, 1999; Loessner et al., 2002). Finally, we have also analysed the expression levels of the *tnp* in S45246, which still possessed the final copy of IS*Pap1*, compared to GR2, deprived of all the copies. As expected, a significant decrease of the expression levels was observed for S45246 compared to no expression at all for GR2. This suggests the full elimination of the IS activity in the genome.

### PCR amplification to track remaining IS*Pap1* copies after IS*Pap1* removal in the genome-reduced strain GR2

To further ensure that no jumping activity of ISPap1 occurred during the construction of GR2, the absence of the IS element in the genome of the respective strain was verified by PCR using primers annealing to a conserved region of the element. As given in Supplementary Figure S2, PCR control on GR2 did not result in any amplicon, indicating that all copies of the transposase were deleted. Genomic DNA of the WT and S45246 were used as positive controls, clearly indicating the expected fragment of 977 bp.

### Growth and metabolite characterization of genome-reduced strains

**FIGURE 5:**
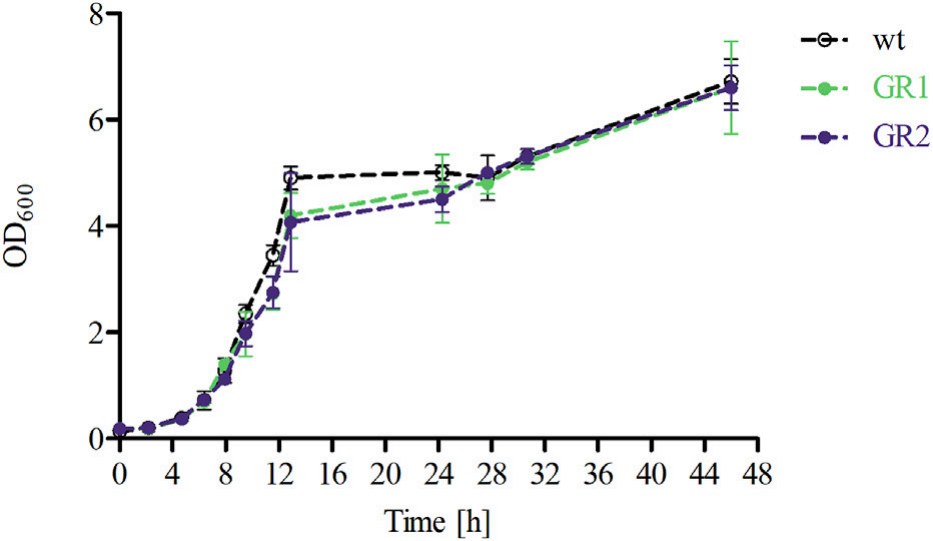
Growth profiles of *P. polymyxa* DSM 365 WT and the genome-reduced strains (GR1 and GR2) during the course of the 48 h of controlled batch cultivation in DASGIP bioreactors applying microaerobic conditions (0.5 vvm).

To further characterise the two genome-reduced strain variants, both the cell growth and the metabolic capacity were analysed. The growth profiles of GR1 and GR2 were assessed by following the optical density OD_600_ and CDW of the cells cultured in fermentation medium in a bioreactor setting. A similar pattern was observed for all strains to the WT (Figure 5; Table 4). The μ_max_ of the variants showed no significant differences with an average of 0.32 ± 0.02 (Table 4).

**TABLE 4:** Overview of process characteristics of *P. polymyxa* DSM 365 WT and the genome-reduced strains. Data are given for 48 h batch fermentations (n = 2).

Strain	μ_max_ [h^-1^]	Y_P/S_ [g g^-1^]	Productivity [g L^-1^ h^-1^]	CDW [g L^-1^]	2,3-BDL [g L^-1^]
WT	0.32 ± 0.02	0.42 ± 0.02	0.40 ± 0.02	2.80 ± 0.16	18.44 ± 1.10
GR1	0.32 ± 0.01	0.42 ± 0.01	0.39 ± 0.02	2.96 ± 0.21	19.76 ± 1.07
GR2	1.31 ± 0.01	0.38 ± 0.02	1.43 ± 0.03	2.78 ± 0.08	17.33 ± 0.89

**FIGURE 6:**
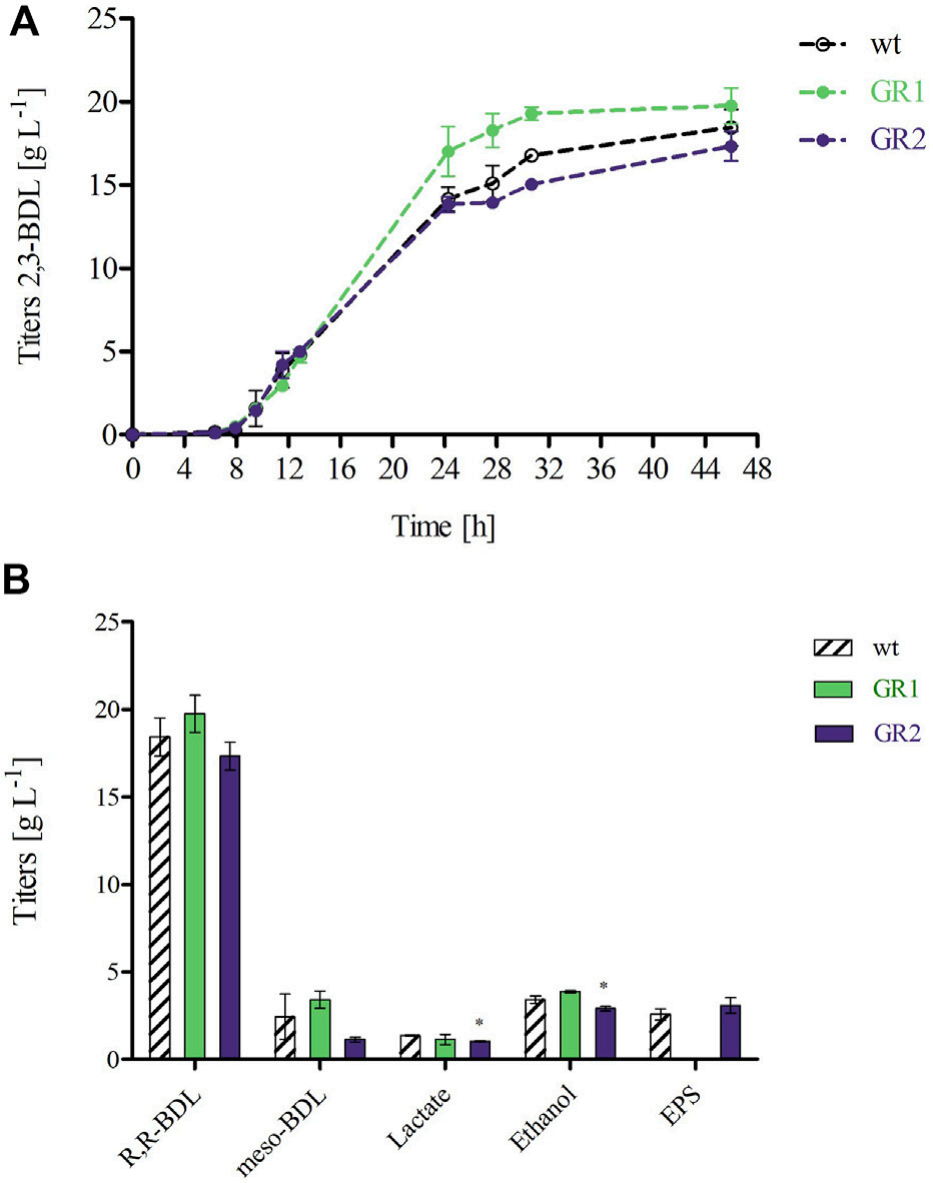
**(A)** 2,3-BDL production over the course of the 48 h fermentation. **(B)** Product profiles of *P. polymyxa* DSM 365 WT and the genome-reduced strains (GR1 and GR2) after 48 h of controlled batch cultivation in the 1 L parallel DASGIP bioreactors under microaerobic conditions (0.5 vvm). Product titers are the result of two independent fermentation processes, *p < 0.05 was determined via two sample t-test.

The production of 2,3-BDL was chosen as a performance indicator for product formation. All strains showed a quite similar 2,3-BDL production within the exponential phase. At 24 h, GR1 reached a 1.2-fold higher titer compared to both the WT and GR2, indicating that the deletion of the selected BGCs caused fluctuations in the metabolism (Figure 6A). Previous studies have shown a correlation between 2,3-BDL and lipopeptide production. Pyruvate can be either directed towards the fermentation products such as of 2,3-BDL or diverted towards acetyl-CoA for fatty acids synthesis required for lipopeptides production, such as polymyxin (Supplementary Figure S3) (Dhali et al., 2017; Yuan et al., 2020). Yuan et al. demonstrated that 2,3-butanediol could be produced at higher titers by a less polymyxin producing mutant of *P. polymyxa* CJX518 (Yuan et al., 2020). GR1 presents the deletion of several BGCs, including two known lipopeptides (*Δphl* and *Δfus*) and possibly two others (*Δupks2* and *Δupks3*), which might have influenced 2,3-BDL production (Li et al., 2007; Vater et al., 2018). However, under the selected conditions, the overall titer and productivity of 2,3-BDL remained unaffected by the genome-reduced variants (Figure 6B). Several studies have shown that the sequential deletion of antibiotic clusters can improve the production of native and/or heterologous antibiotics by redirecting the precursor’s supply (Myronovskyi et al., 2018; Ahmed et al., 2020; Zhang et al., 2020). Therefore, although we have not observed any effect on the production of 2,3-BDL, the deletion of these BGCs in GR1 might have an effect on antibiotic production. This prospect should be explored by further research.

By the 2,3-BDL pathway only 1 mol of NADH is converted to NAD^+^ whereas 2 mols of NADH are formed during glycolysis. Therefore, to maintain redox balance, other redox neutral end products such as lactate or ethanol compete for the intermediate pyruvate and are formed via the fermentative pathway (Supplementary Figure S3) (Ji et al., 2011). In our experiment, all strains produced meso-BDL, lactate and ethanol, but no formate. Interestingly, GR2 showed significantly lower lactate and ethanol titers (1.4-fold and 1.2-fold, respectively). Owing to the deletion of several hypothetical proteins within the GIs, a conclusive explanation for this significant difference cannot be given. However, this discrepancy raises a topic for discussion.

In addition, we have examined EPS production at the end of the fermentation process. As expected, no EPS production was observed for GR1. Whereas GR2 maintained unaltered EPS titers compared to the WT (3.08 ± 0.45 and 2.57 ± 0.32 g L^-1^, respectively) as given in Figure 6B. Schilling et al. characterized *P. polymyxa* DSM 365 under similar conditions, employing a distinct aeration input of 0.075 vvm and extending the duration of the fermentation to 72 h (Schilling et al., 2020). We observed a comparable 2,3-BDL titer at 48 h of 18.44 ± 1.10 g L^-1^, with a corresponding productivity of 0.40 ± 0.02 g L^-1^ h^-1^. Notably, higher oxygen levels led to the production of meso-BDL, reduced lactate, no formate and increased EPS productions (Nakashimada et al., 1998; Rütering et al., 2016).

## Conclusion

In our present study, we were able to close the sequence gaps of the genome of *P. polymyxa* DSM 365 with 5,889,536 bp, which is the prerequisite for further development of this promising non-model organism. Thereby, we identified an unknown NRPS for which we hypothesise a possible role in tridecaptin’s production, thus, providing new directions for future studies. We have also elucidated the antibiotic production capacity of this strain, with still four unknown BGCs. By specific knockout experiments, we have shown that the polymyxin and tridecaptin cluster are not essential but might be significantly affect *P. polymyxa*´s growth. In contrast we have also shown that several hypothetical proteins which are located in G1 and G2 do not have an impact on fitness. By deleting 178.5 kb and 38.5 kb we created two genome-reduced strains (GR1 and GR2). Although, no enhancement in 2,3-BDL formation was shown, both the growth characteristics and the desired product formation of the two strains were maintained at WT-level, an extremely important achievement and starting point for future targeted genetic modifications.

## Data Availability

The annotated genome sequence of *P. polymyxa* DSM 365 has been deposited in GenBank under the BioProject accession number PRJNA1051977, the BioSample accession number SAMN38818135. The complete genome sequence has been deposited under the accession number CP141264.
