# Corrigendum: Genome reduction in *Paenibacillus polymyxa* DSM 365 for chassis development

**DOI:** 10.3389/fbioe.2024.1451723

**Published:** 2024-07-04

**Authors:** Giulia Ravagnan, Janne Lesemann, Moritz-Fabian Müller, Anja Poehlein, Rolf Daniel, Stephan Noack, Johannes Kabisch, Jochen Schmid

**Affiliations:** ^1^ Institute of Molecular Microbiology and Biotechnology, University of Münster, Münster, Germany; ^2^ Institute of Bio- and Geosciences, IBG-1: Biotechnology, Forschungszentrum Jülich GmbH, Jülich, Germany; ^3^ Department of Genomic and Applied Microbiology and Göttingen Genomics Laboratory, Institute of Microbiology and Genetics, Georg-August-University Göttingen, Göttingen, Germany; ^4^ Department of Biotechnology and Food Science, Norwegian University of Science and Technology, Trondheim, Norway

**Keywords:** *Paenibacillus polymyxa*, genome reduction, chassis, BGCS, 2,3-butanediol

In the published article, there was an error in the **Funding** statement. The funder Deutsche Forschungsgemeinschaft (DFG, German Research Foundation), (No. 491062903) was not included. The correct **Funding** statement appears below.

