# Supplementary figures and images for: Genome reduction in Paenibacillus polymyxa DSM 365 for chassis development
Source: Front Bioeng Biotechnol. 2024 Mar 28;12:1378873. doi: 10.3389/fbioe.2024.1378873 (PMC11007031; doi:10.3389/fbioe.2024.1378873)

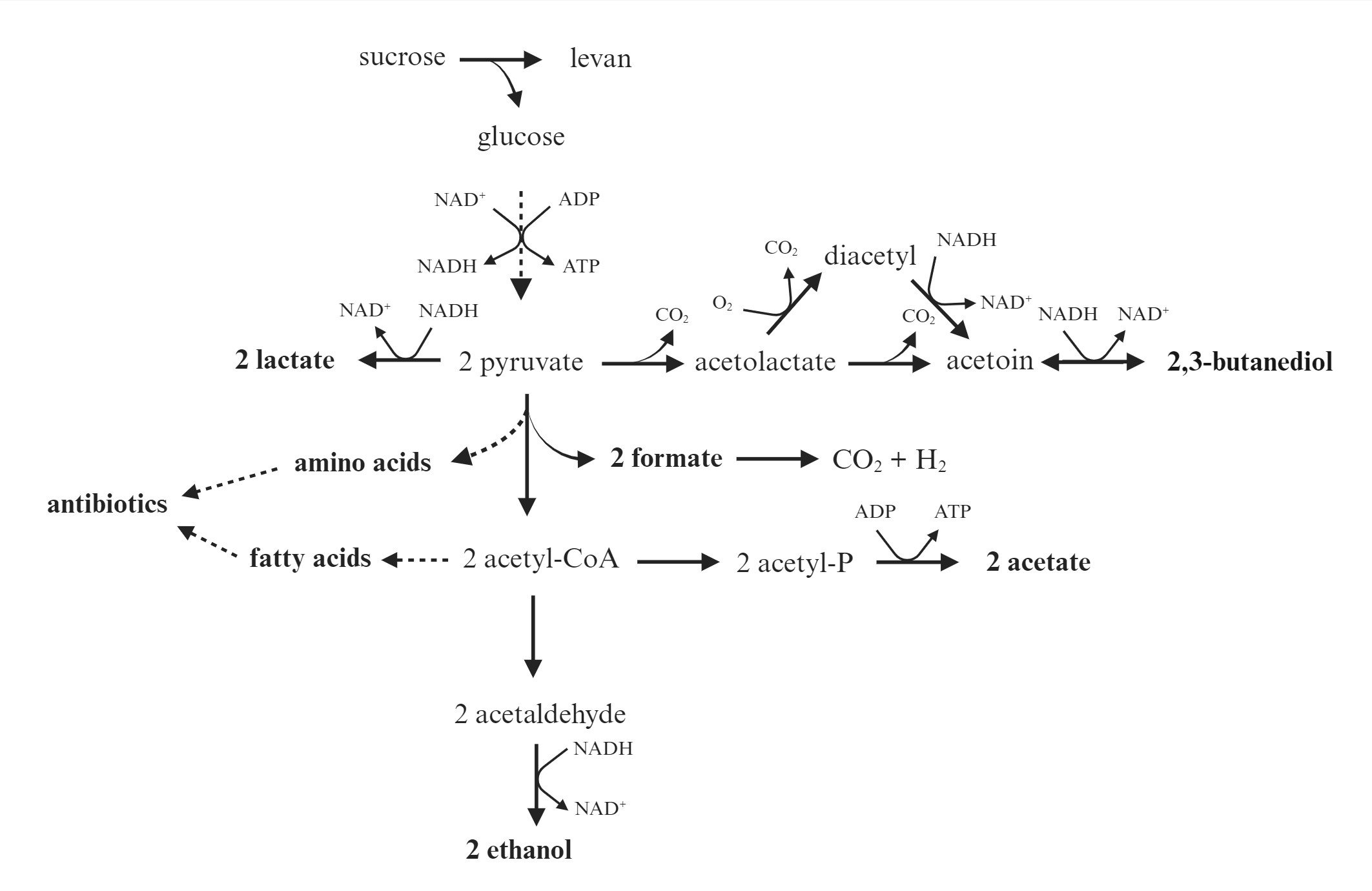

Supplement: Supplementary file 1 [file Image3.jpeg]

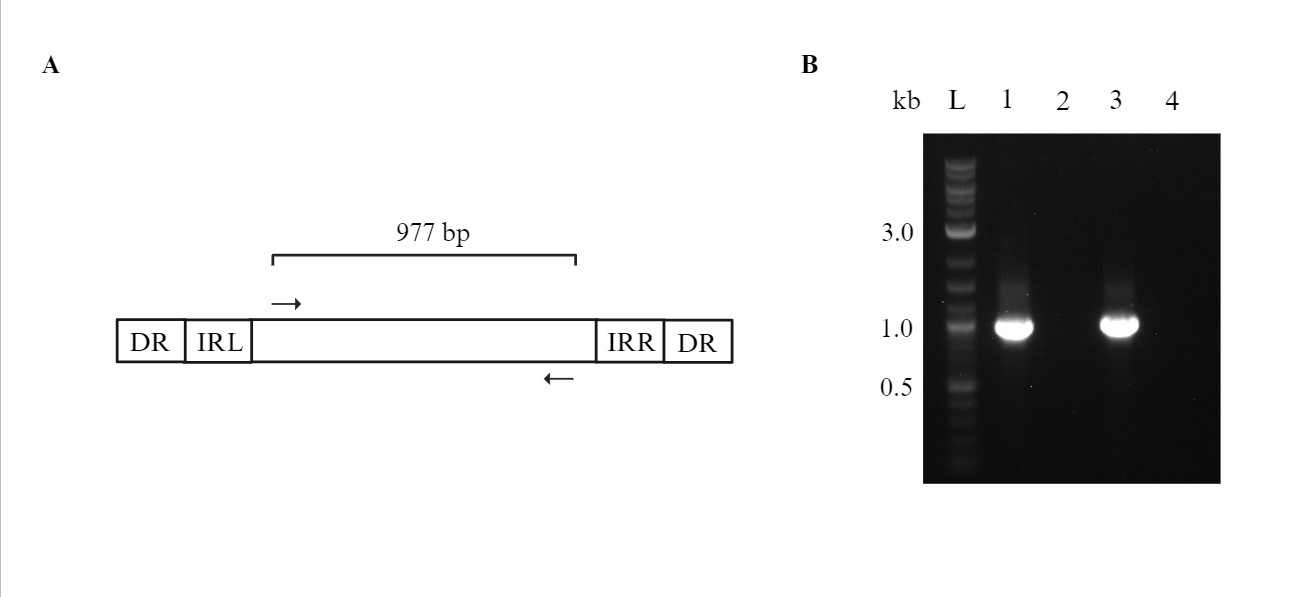

Supplement: Supplementary file 2 [file Image2.jpeg]

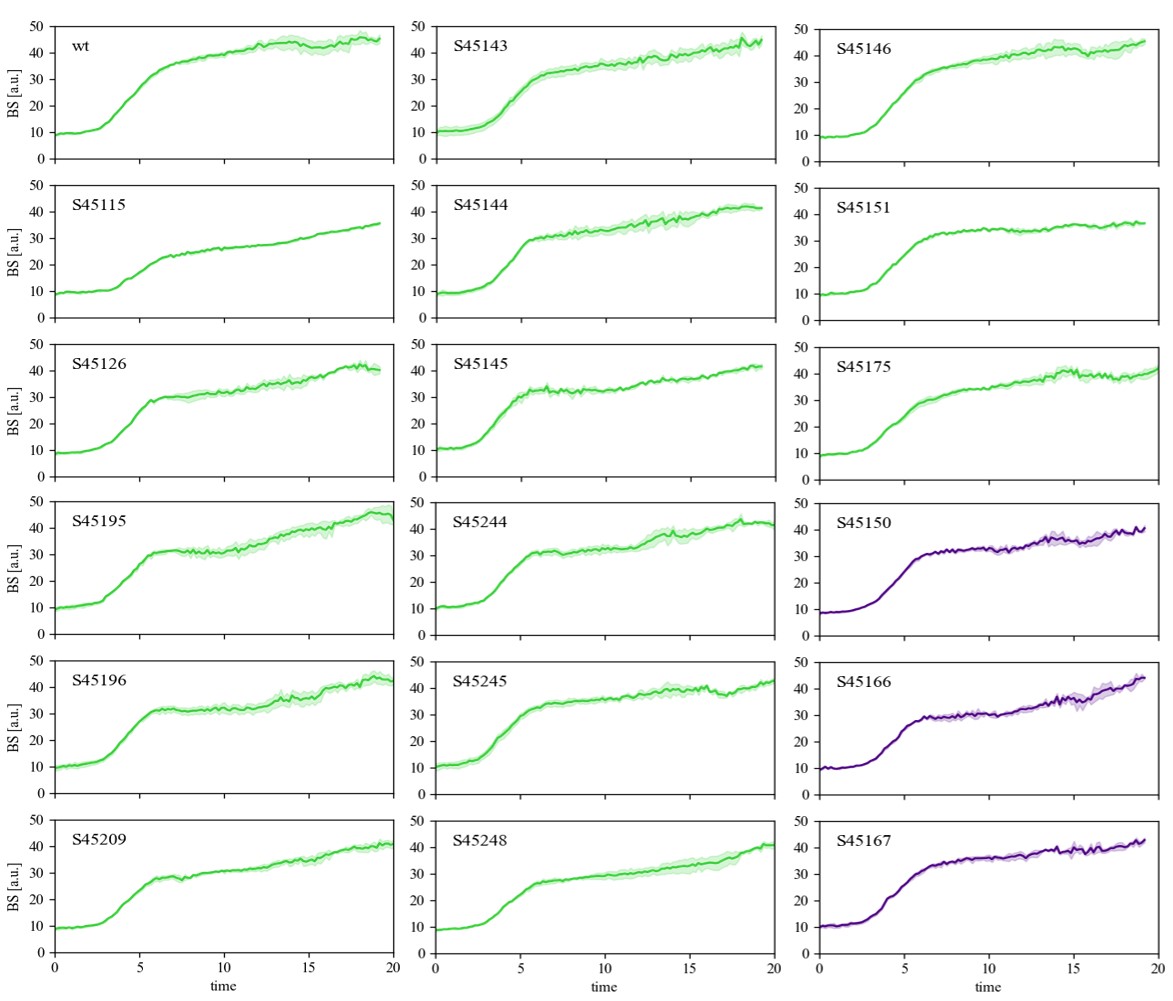

Supplement: Supplementary file 4 [file Image1.jpg]
