# Supplementary material for: Genome reduction in Paenibacillus polymyxa DSM 365 for chassis development
Source: Front Bioeng Biotechnol. 2024 Mar 28;12:1378873. doi: 10.3389/fbioe.2024.1378873 (PMC11007031; doi:10.3389/fbioe.2024.1378873)
Supplement: Supplementary file 3 [file DataSheet1.PDF]

Supplementary Material

Supplementary Figures and Tables

Supplementary Table S1. List of primers used in this study.

Primer	Sequence (5´-3´)	Purpose
pCasPP_bb2_fw	TCTAGTGGCCAGGAACCGTAAAAAGG	Amplification of pCasPP backbone
pCasPP_bb3_rev	CGACATTGATATGAATATGCCTGTAACAG	Amplification of pCasPP backbone
pCasPP_bb2_fw	CTGTTACAGGCATATTCATATCAATGTCG	Amplification of pCasPP backbone
pCasPP_bb2_rv	GCGTATCCCCTTTCAGATACTCGC	Amplification of pCasPP backbone
oriT_rev	CTAGTCGGCGGGCTTGATGC	Amplification of pCasPP backbone
fus_check_fw	TATGCCATTCGTCCACGCTC	Screening of *fus* deletion
fus_check_rv	CATCTAAGAACGCCGTGAGATC	Screening of *fus* deletion
paen_sgRNA_fw	GTATCTGAAAGGGGATACGCAATATCCAC TATAATTACAGGTTTTAGAGCTAGAAATA G	Construction of pCasPP-paen
paen_uhs_fw	GCATCAAGCCCGCCGACTAGGGCGATATG GCAATCAAG	Construction of pCasPP-paen
paen_uhs_rv	GGTGTCTCGTATAAATCGG	Construction of pCasPP-paen
paen_dhs_fw	GCCGATTTATACGAGACACCGACGGGAAA CGACATGTT	Construction of pCasPP-paen

paen_dhs_rv	TACGGTTCCTGGCCACTAGATAAAACGTCCCTGCGCCA	Construction of pCasPP-paen
paen_ext_fw	CTTAACGACATGCTCAGG	Screening of *paen* deletion
paen_ext2_rv	GGGACCAAAGTGATTCGA	Screening of *paen* deletion
sgRNA_pnl_fw	GTATCTGAAAGGGGATACGCTGCTAAGAAAGCAACCTGTGGTTTTAGAGCTAGAAATAG	Construction of pCasPP-pnl
pnl_uhs_fw	GCATCAAGCCCGCCGACTAGCTCTTCCGTTATCAGATCG	Construction of pCasPP-pnl
pnl_uhs_rv	CCTCATTCCTCCATCTTG	Construction of pCasPP-pnl
pnl_dhs_fw	GTCAAGATGGAGGAATGAGGTCGCTCTCGTTAGTAAGG	Construction of pCasPP-pnl
pnl_dhs_rv	TACGGTTCCTGGCCACTAGACCTCATCAGGGGTTAGCA	Construction of pCasPP-pnl
pnl_ext_fw	TGTATGTATTGGGTTCGC	Screening of *pnl* deletion
pnl_ext_rv	ATAATCTGTTGACCGTCC	Screening of *pnl* deletion
sacB_KOproof_F	AATTATCGCATTGCTGCCCAGACAG	Screening of *sacB* deletion
sacB_KOproof_R	AGATCGGGTTGCTACCAATCTACCG	Screening of *sacB* deletion
gum_int_check_fw	ATCCGTCATGGATTGGCCAAG	Screening of *pep* deletion
clu1clu2_KO_check_rev	CGGATGATACATCGCATTCG	Screening of *pep* deletion
bb_inpro_rv	CCGTCGACCCATAAGTCCACGCGTATCCCCTTTCAGATACTCG	Construction of pCasPP-pro
sgRNA_inpro_fw	GTGGACTTATGGGTCGACGGGTTTTAGAGCTAGAAATAGCAAGTTAAAATAAGGC	Construction of pCasPP-pro

inpro_uhs_fw	GCATCAAGCCCGCCGACTAGAAGAGAGCTTGCCGAACG	Construction of pCasPP-pro
inpro_uhs_rv	GATGAAATGCAACAGCGGCAACAGTCCTTTTGGCAATC	Construction of pCasPP-pro
inpro_dhs_fw	TGCCGCTGTTGCATTTCA	Construction of pCasPP-pro
inpro_dhs_rv	TACGGTTCCTGGCCACTAGAGTTTTCCCTGCCTCTGGC	Construction of pCasPP-pro
inpro_ext_fw	TGCACGCCCTCTCCTGCT	Screening of *pro* deletion
inpro_ext_rv	GGCAAATCCAGGGATATTG	Screening of *pro* deletion
sgRNA_unrps_2_fw	GTATCTGAAAGGGGATACGCTAGCAACACCATGTATAAGGGTTTTAGAGCTAGAAATAG	Construction of pCasPP-unrps
uhs_unrps_3_fw	GCATCAAGCCCGCCGACTAGCTAAAAGACGTGATATACGATTG	Construction of pCasPP-unrps
uhs_unrps_3_rv	CACATCAAGCTGAAGTGA	Construction of pCasPP-unrps
dhs_unrps_3_fw	AATCACTTCAGCTTGATGTGTCTTAATTACAAAGGCTATAAAAC	Construction of pCasPP-unrps
dhs_unrps_3_rv	TACGGTTCCTGGCCACTAGACATACAAGAATTTACTAGAAC	Construction of pCasPP-unrps
ext_unrps_fw	ATTTTGCACCCATTCCAG	Screening of *urnps* deletion
ext_unrps_rv	GCAAGTCTACGTCATTTTG	Screening of *urnps* deletion
sgRNA_GI_fw	GTATCTGAAAGGGGATACGCCTATATAGGTTATAGCGGAGGTTTTAGAGCTAGAAATAG	Construction of pCasPP-GI
uhs_GI_fw	GCATCAAGCCCGCCGACTAGCTGCCCTTTCCTAGAGCA	Construction of pCasPP-GI
uhs_GI_rv	TTGCCACCCATGCTAGAA	Construction of pCasPP-GI

dhs_GI_fw	TATTCTAGCATGGGTGGCAATGGAGTTGATATAGTTTATCAACTC	Construction of pCasPP-GI
dhs_GI_rv	TACGGTTCCTGGCCACTAGAAAATCAAAAGGCCGGCAC	Construction of pCasPP-GI
ext_GI_fw	ATCATCTATGCGCATAGG	Screening of *GI* deletion
ext_GI_rv	CGTTTCTTACTGCGCTTG	Screening of *GI* deletion
sgRNA_GI_2_fw	GTATCTGAAAGGGGATACGCAAAGTCACATCCGTTTCGAGGTTTTAGAGCTAGAAATAG	Construction of pCasPP-GI2
uhs_GI_2_fw	GCATCAAGCCCGCCGACTAGGCAACCAAACACAGCCAG	Construction of pCasPP-GI2
uhs_GI_2_rv	CTGTTTCTTTGGCATCGG	Construction of pCasPP-GI2
dhs_GI_2_fw	GGCCGATGCCAAAGAAACAGTGATTTGTGTCGAAGAGAT	Construction of pCasPP-GI2
dhs_GI_2_rv	TACGGTTCCTGGCCACTAGACTAGCGCTTTTCAGCGTA	Construction of pCasPP-GI2
ext_GI_2_fw	CTGATGCCAAATCATCCG	Screening of *GI2* deletion
ext_GI_2_rv	CCAAGAGAGCCAACTAGC	Screening of *GI2* deletion
sgRNA_pks2_fw	GTATCTGAAAGGGGATACGCCGAAAAAGTGTACCGCACAGGTTTTAGAGCTAGAAATAG	Construction of pCasPP-pks2
uhs_pks2_fw	GCATCAAGCCCGCCGACTAGCCAAACTTCTGGAGCTAG	Construction of pCasPP-pks2
uhs_pks2_rv	GCCCAATCCACATCCATG	Construction of pCasPP-pks2
dhs_pks2_fw	AACATGGATGTGGATTGGGCCTAAAACAGGCATTCTCTC	Construction of pCasPP-pks2

dhs_pks2_rv	TACGGTTCCTGGCCACTAGAGTTGAGGAACTTGCACTC	Construction of pCasPP-pks2
ext_pks2_fw	TGCTGAGCTCGATATTAC	Screening of *pks2* deletion
ext_pks2_rv	AATCCGTCCGATCCTCAT	Screening of *pks2* deletion
bb_tridecaptin_rv	ATGTCATCTATACCTCCGGCGCGTATCCCCTTTCAGATACTCG	Construction of pCasPP-tri
sgRNA_tridecaptin_fw	GCCGGAGGTATAGATGACATGTTTTAGAGCTAGAAATAGCAAGTTAAAATAAGGC	Construction of pCasPP-tri
uhs_tri2_fw	GCATCAAGCCCGCCGACTAGAAAAACTCAATCCAAACTGG	Construction of pCasPP-tri
tridecaptin_uhs_rv	ATGTTCAAGATTTATGATGTCCTCTATGCAGTTCATAATTTTAC	Construction of pCasPP-tri
tridecaptin_dhs_fw	ACATCATAAATCTTGAACATCTATG	Construction of pCasPP-tri
tridecaptin_dhs_rv	TACGGTTCCTGGCCACTAGATTAACGGCAATTCCAGCGT	Construction of pCasPP-tri
tridecaptin_ext1_fw	CTGTTTCAAATACGCTCC	Screening of *tri* deletion
tridecaptin_ext2_rv	GGAAGTTGTATAGGCAAC	Screening of *tri* deletion
sgRNA_upks_fw	GTATCTGAAAGGGGATACGCTCACAGGGACAAGTTCCTGGGTTTTAGAGCTAGAAATAG	Construction of pCasPP-upks
uhs_upks_fw	GCATCAAGCCCGCCGACTAGAGGTGTCGAAATTTGTATG	Construction of pCasPP-upks
uhs_upks_rv	ATACGCTTCCTGATCAAC	Construction of pCasPP-upks
dhs_upks_fw	AGGTTGATCAGGAAGCGTATGGCTTTAAATCCTAGGAC	Construction of pCasPP-upks

dhs_upks_rv	TACGGTTCCTGGCCACTAGATAACTTCTGCATCCAGTG	Construction of pCasPP-upks
ext_upks_fw	TGATGGACCATCCTGTTC	Screening of *upks* deletion
ext_upks_rv	TGGCATCAAGAAGTCCAC	Screening of *upks* deletion
sgRNA_upks3_fw	ACGCAGTTCATGGAATCGAAAACG	Construction of pCasPP-upks3
sgRNA_upks3_rv	AAACCGTTTTCGATTCCATGAACT	Construction of pCasPP-upks3
uhs_upks3_fw	GCATCAAGCCCGCCGACTAGCAGTTGGGTGTCCCTGCG	Construction of pCasPP-upks3
uhs_upks3_rv	CTCCCCACAAAGATGTGG	Construction of pCasPP-upks3
dhs_upks3_fw	TGCCACATCTTTGTGGGGAGGCCATGTGCCAATGCCAG	Construction of pCasPP-upks3
dhs_upks3_rv	TACGGTTCCTGGCCACTAGACCGAAGCTGGTTTATAGAAA	Construction of pCasPP-upks3
ext_upks3_fw	AACTCTCCATTAAAGACCAA	Screening for *upks3* deletion
ext_upks3_rv	ACGGGAGAAAACGAATTG	Screening for *upks3* deletion
sgRNA_phl_4_rv	GTATCTGAAAGGGGATACGCTTAACAGCCGAACGTCACGGGTTTTAGAGCTAGAAATAG	Construction of pCasPP-phl
uhs_phl_4_fw	GCATCAAGCCCGCCGACTAGCAAGAAACGAAGTGACTTCTTG	Construction of pCasPP-phl
uhs_phl_4_rv	GCGAGGATCTAATTTGTTTTATTAAAC	Construction of pCasPP-phl
dhs_phl_4_fw	AAAACAAATTAGATCCTCGCGCAGGCTGTACGTGGAGA	Construction of pCasPP-phl
dhs_phl_4_rv	TACGGTTCCTGGCCACTAGAGCATCTCCTTCATCCGTT	Construction of pCasPP-phl

ext_phl_4_fw	CAGATGCCGTACCCAATC	Screening of *phl* deletion
ext_phl_4_rv	GGATCAAAGGCAGTTCTG	Screening of *phl* deletion
sgRNA_thd_fw	GTATCTGAAAGGGGATACGCGTAATTCAAGGTCACCCTGAGTTTTAGAGCTAGAAATAG	Construction of pCasPP-thd
uhs_thd_fw	GCATCAAGCCCGCCGACTAGCTCCATACCCACCTTAGA	Construction of pCasPP-thd
uhs_thd_rv	GCTTCACTTCATCTCCTT	Construction of pCasPP-thd
dhs_thd_fw	AGAAGGAGATGAAGTGAAGCGGAGGGGAGCTTCAGATT	Construction of pCasPP-thd
dhs_thd_rv	TACGGTTCCTGGCCACTAGAAGTCGGGTAAGAAAGGCA	Construction of pCasPP-thd
ext_thd_fw	GAATTCCCCTGAAGCGTC	Screening of *thd* deletion
ext_thd_rv	AATCCAGCTGGCTCCTCC	Screening of *thd* deletion
sgRNA_pae_4_fw	GTATCTGAAAGGGGATACGCAGCTGTGTGTCCAAACCTTGGTTTTAGAGCTAGAAATAG	Construction of pCasPP-pae
pae_uhs_3_fw	GCATCAAGCCCGCCGACTAGCGTGGCTTATGATGAGTG	Construction of pCasPP-pae
pae_uhs_3_rv	AGGGAATAACATGACATATCGTTCTAAGCCAAACGTTTA	Construction of pCasPP-pae
pae_dhs_fw	GATATGTCATGTTATTCCCTAAC	Construction of pCasPP-pae
dhs_pae_2_rv	TACGGTTCCTGGCCACTAGAGGATTGACCTGTGTCACT	Construction of pCasPP-pae
ext_pae_fw	TGTCTTATTCCGTATTTCC	Screening of *pae* deletion
pae_ext_rv	CTCTGGCTTCAACAAAAAG	Screening of *pae* deletion

sgRNA_tnp6_1_fw	GAGACATCTTTGAAGACAAACGCTTTCTCGACAGCCTGAAGGGGTTTTAGAGCTAGAAATAGCAAG	Construction of pCasPP-ISPap1-1
uhs_tnp6_1_fw	GCATCAAGCCCGCCGACTAGAGTGCGGGAGATACGTTA	Construction of pCasPP-ISPap1-1
uhs_tnp6_1_rv	TTTTGTATTAATTTGATTTGTATTTATCAAG	Construction of pCasPP-ISPap1-1
dhs_tnp6_1_fw	CAAATCAAATTAATACAAAATGAAGGGCGGAGTTTTAC	Construction of pCasPP-ISPap1-1
dhs_tnp6_1_rv	TACGGTTCCTGGCCACTAGAGATAGTGGCAGTTTGGTATT	Construction of pCasPP-ISPap1-1
ext_tnp6_1_fw	GCGACTCCATCAAATCCAC	Screening of IS*Pap1-1* deletion
ext_tnp6_1_rv	GCGTGATATATCGAGCGC	Screening of IS*Pap1-1* deletion
sgRNA_tnp6_3_fw	GTATCTGAAAGGGGATACGCAAAAAAAGGACCCTATCCGAGTTTTAGAGCTAGAAATAG	Construction of pCasPP-ISPap1-3
uhs_tnp6_3_2_fw	GCATCAAGCCCGCCGACTAGGGTTTGCAACTCATAACC	Construction of pCasPP-ISPap1-3
uhs_tnp6_3_rv	CTGAGGACCCTATCCGAG	Construction of pCasPP-ISPap1-3
dhs_tnp6_3_fw	CCCTCGGATAGGGTCCTCAGCCCTGTCTCAGGATTCCT	Construction of pCasPP-ISPap1-3
dhs_tnp6_3_rv	GGATAAAGGTAAAAAGAGCAAA	Construction of pCasPP-ISPap1-3
ext_tnp6_3_fw	CCTGGTTATTCGCAGGTT	Screening of IS*Pap1-3* deletion
ext_tnp6_3_rv	CTGCTGATCCAGTATCCA	Screening of IS*Pap1-3* deletion

sgRNA_tnp6_5_fw	GTATCTGAAAGGGGATACGCAAGGTCGCCTCATAAAGAGGGTTTTAGAGCTAGAAATAG	Construction of pCasPP-ISPap1-5
uhs_tnp6_5_fw	GCATCAAGCCCGCCGACTAGATCTGGTACTGGAACCCG	Construction of pCasPP-ISPap1-5
uhs_tnp6_5_rv	TATGCATACATACTCTTAAGCAA	Construction of pCasPP-ISPap1-5
dhs_tnp6_5_fw	CTTAAGAGTATGTATGCATAGGCGGCTTTTTTTTGTGTTTT	Construction of pCasPP-ISPap1-5
dhs_tnp6_5_rv	TACGGTTCCTGGCCACTAGATACGTCCGCGCCATTCCT	Construction of pCasPP-ISPap1-5
ext_tnp6_5_fw	CTCCAGCAGCTCTACCAG	Screening of IS*Pap1-5* deletion
ext_tnp6_5_rv	CCGCAAAATGTCAGAGCT	Screening of IS*Pap1-5* deletion
sgRNA_tnp6_6_2_fw	GTATCTGAAAGGGGATACGCGGGTACACTGACTTAGCAGTGTTTTAGAGCTAGAAATAG	Construction of pCasPP-ISPap1-6
uhs_tnp6_6_2_fw	GCATCAAGCCCGCCGACTAGTTAATCGTTCTCTCCTTGTTT	Construction of pCasPP-ISPap1-6
uhs_tnp6_6_2_rv	TTGACAAAATAACTTGTTGTTC	Construction of pCasPP-ISPap1-6
dhs_tnp6_2_fw	ACAACAAGTTATTTTGTCAACTGGCGAATTAAGCTTTTGG	Construction of pCasPP-ISPap1-6
dhs_tnp6_2_rv	TACGGTTCCTGGCCACTAGAGGTCCCGTAAGTGCTGAG	Construction of pCasPP-ISPap1-6
ext_tnp6_6_fw	CATATTTAGGGTGACGCC	Screening of IS*Pap1-6* deletion
ext_tnp6_6_rv	CAATCGACATCGCTAGAC	Screening of IS*Pap1-6* deletion
tnp6_check_2_fw	CACTCTTTGCTGTCTTCC	Screening of a conserved region of IS*Pap1*, to check absence of

		other gene copies in the genome of GR2
tnp6_check_2_rv	CCATTGATCCTACCGTATTG	Screening of a conserved region of IS*Pap1*, to check absence of other gene copies in the genome of GR2
gyrA_qpcr_fw	GAGATATGGCCGCTGCGATG	qPCR primer for *gyrA*
gyrA_qpcr_rv	GCTCTCTTCACCATCGTAGTTCGG	qPCR primer for *gyrA*
orfA_tnp6_qpcr_fw	GATCTGGTTCCTCAAGATCAC	qPCR primer for IS*Pap1*
orfA_tnp6_qpcr_rv	GTAGGATCAATGGCAGGAC	qPCR primer for IS*Pap1*

Supplementary Table S2. List of plasmids used in this study.

**Plasmids**	**Description**	**Source or reference**
pCasPP	*P. polymyxa* CRISPR-Cas9 genome editing plasmid	(Rütering et al., 2017)
pCasPP-fussg2	Deletion of fusaricidin	(Meliawati et al., 2022b)
pCasPP-paen	Deletion of paenibacillin	This study
pCasPP-pnl	Deletion of paenilan	This study
pCasPP-pep	Deletion of paenan	(Meliawati et al., 2022b)
pCasPP-sacB	Deletion of levansucrase	(Schilling et al., 2020)
pCasPP-pro	Deletion of the incomplete prophage	This study
pCasPP-unrps	Deletion of unknown NRPS	This study

pCasPP-GI1	Deletion of genomic island 1	This study
pCasPP-GI2	Deletion of genomic island 2	This study
pCasPP-upks2	Deletion of unknown pks 2	This study
pCasPP-tri	Deletion of tridecaptin	This study
pCasPP-upks	Deletion of unknown pks	This study
pCasPP-upks3	Deletion of unknown pks 3	This study
pCasPP-phl	Deletion of a part of paenilipoheptin	This study
pCasPP-thd	Deletion of thermoactinoamide	This study
pCasPP-pae	Deletion of a part of paenicidin	This study
pCasPP-ISPap1-1	Deletion of ISPap1-1, first copy of ISPap1	This study
pCasPP-ISPap1-3	Deletion of ISPap1-3, third copy of ISPap1	This study
pCasPP-ISPap1-5	Deletion of ISPap1-5, fifth copy of ISPap1	This study
pCasPP-ISPap1-6	Deletion of ISPap1-6, sixth copy of ISPap1	This study

Supplementary Table S3. Detailed information of all the deleted genomic regions in *P. polymyxa* DSM 365.

**Genes or gene clusters deleted**	**Coordinates deleted (bp)**		**Deletion size (bp)**
	**Start**	**End**	
Δ*pmx*	1,246,011	1,287,231	41,220
Δ*fus*	327,498	358,533	31,035

Δ*paen*	5,288,491	5,300,391	11,900
Δ*pnl*	4,612,625	4,625,065	12,473
Δ*pep*	4,956,602	4,989,448	32,846
Δ*sacB*	5,524,870	5,526,464	1,595
Δ*pro*	5,013,700	5,033,790	20,091
Δ*unrps*	3,477,388	3,520,882	43,494
Δ*GI1*	2,401,200	2,420,275	19,075
Δ*GI2*	3,368,965	3,711,912	13,016
Δ*upks2*	3,350,629	3,390,854	40,225
Δ*tri*	3,777,769	3,836,249	58,481
Δ*upks*	1,928,884	1,977,283	48,400
Δ*upks3*	3,626,698	3,651,242	24,545
Δ*phl*	3,557,020	3,574,407	17,388
Δ*thd*	3,238,677	3,250,671	11,994
Δ*pae*	760,922	767,379	6,456
Δ*ISPap1-1*	117,205	118,782	1,578
Δ*ISPap1-3*	3,149,211	3,150,799	1,588
Δ*ISPap1-5*	4,120,779	4,122,376	1,597
Δ*ISPap1-6*	4,264,668	4,266,344	1,677

Supplementary Figure S1. Growth profiles of *P. polymyxa* DSM 365 WT and the single deletion variants, measured by culturing in CASO Broth medium in the BioLector (n ≥ 4). The growth of the strains were monitored in a 48-well FlowerPlate by measuring backscatter (BS, gain 20).

Supplementary Figure S2. (A) Genetic representation of IS*Pap1* indicating the positions of the forward (→) and reverse (←) primers in the conserved region. The boxes at each end represent the direct repeat (DR), the left (IRL) and right (IRR) terminal inverted repeats. (B) Gel electrophoresis

showing PCR fragments generated upon PCR amplification with GoTaq from the purified gDNA of S45246 (lane 1), GR2 (lane 2), WT (lane 3) and a negative control (lane 4), respectively.

Supplementary Figure S3. Overview of the mixed acid pathway and butanediol biosynthesis of *Paenibacillus polymyxa*. Two mols of NADH are formed during glycolysis that need to be regenerated in order to maintain redox balance. Only 1 mol NADH is converted to NAD^+ in the 2,3-BDL pathway. Therefore, other redox neutral end products such as lactate or ethanol compete for the intermediate pyruvate. Pyruvate is also used for the production of antibiotics. Furthermore, sucrose and glucose are utilized for the production of the two exopolysaccharides levan and paenan.
